# Standalone Effects of a Cognitive Behavioral Intervention Using a Mobile Phone App on Psychological Distress and Alcohol Consumption Among Japanese Workers: Pilot Nonrandomized Controlled Trial

**DOI:** 10.2196/mental.8984

**Published:** 2018-03-22

**Authors:** Toshitaka Hamamura, Shinichiro Suganuma, Mami Ueda, Jack Mearns, Haruhiko Shimoyama

**Affiliations:** ^1^ Division of Clinical Psychology University of Tokyo Bunkyo, Tokyo Japan; ^2^ Japan Society for the Promotion of Science Chiyoda, Tokyo Japan; ^3^ Department of Psychology California State University, Fullerton Fullerton, CA United States

**Keywords:** mobile phone intervention, cognitive behavioral therapy, psychological distress, drinking, Japanese workers

## Abstract

**Background:**

Research that investigates standalone effects of a mobile phone-based cognitive behavioral therapy without any human contact for reducing both psychological distress and risky drinking has been advancing; however, the number of studies is still limited. A mobile phone app called Self Record that facilitates cognitive restructuring through self-monitoring of daily thoughts and activities was developed in Japan.

**Objective:**

This study conducted a nonrandomized controlled pilot trial of the Self Record app to investigate standalone effects of the intervention on psychological distress and alcohol consumption among Japanese workers. Additionally, we examined moderating effects of negative mood regulation expectancies, which are beliefs about one’s ability to control one’s negative mood.

**Methods:**

A quasi-experimental design with a 1-month follow-up was conducted online in Japan from February 2016 to March 2016. A research marketing company recruited participants. The selection criteria were being a Japanese full-time worker (age 20-59 years), experiencing mild to moderate psychological distress, and having some interest in self-record apps. Assignment to group was based on participants’ willingness to use the app in the study. All participants completed outcome measures of negative mood regulation expectancies, positive well-being, general distress, depression, anxiety, and typical/most weekly alcohol consumption.

**Results:**

From the recruitment, 15.65% (1083/6921) of participants met the inclusion criteria. Of these, 51.43% (557/1083) enrolled in the study: 54.9% (306/557) in the intervention group and 45.1% (251/557) in the control group. At the 1-month follow-up, 15.3% (85/557) of participants had dropped out. Intention-to-treat analyses revealed that participants in the intervention group reported increased typical drinking (η2=.009) and heavy drinking (η2=.001). Adherence to using the app was low; 64.8% (199/306) of participants in the intervention group discontinued using the app on the first day. Additionally, 65.7% (366/557) of the total sample did not correctly answer the validity checks in the outcome measures (eg, “Please select ‘mildly agree’ for this item”). Therefore, per-protocol analyses were conducted after removing these participants. Results showed that continuing app users (42/127) in the intervention group reported increases in anxiety (η2=.006), typical drinking (η2=.005), and heavy drinking (η2=.007) compared to those in the control group (85/127). Negative mood regulation expectancies moderated the effects of the intervention for general distress (beta=.39).

**Conclusions:**

Results were contrary to our hypotheses. Self-recording methods of standalone mobile phone interventions may heighten individuals’ awareness of their pathological thought and drinking behavior, but may be insufficient to decrease them unless combined with a more intense or face-to-face intervention. Limitations include high attrition in this study; measures to improve the response rate are discussed.

## Introduction

### Background

Computer-delivered interventions (CDIs) are therapeutic or quasi-therapeutic interactions delivered digitally rather than through interaction with another person. They have become widely available over the last decade and benefits of CDIs include cost-effectiveness and accessibility to a wider population [1]. Recent systematic reviews and meta-analyses have shown these interventions to be effective for treating depression [2] and anxiety [3]. For risky drinking, CDIs appear to have weak effects at 6 months, but none at 12 months [4].

Recent increases in the number of people carrying mobile phones have led to the development of psychological interventions through mobile apps. Evidence-based apps are only a small portion of available apps, but studies have shown that mobile phone-based CDIs have the potential to reduce psychological and physical symptoms [5-7] and possibly risky drinking [8]. In light of their cost-effectiveness and accessibility to many users, mobile phone-based interventions may be useful in a hierarchical model of treatment.

### Cognitive Behavioral Therapy

Cognitive behavioral therapy (CBT) is a widely used psychological intervention and much evidence shows its effectiveness for a number of psychological disorders, including depression, anxiety [9], and substance abuse [10]. An important element of CBT is monitoring one’s thoughts and activities. Daily behavioral and thought records are often assigned as homework to examine individuals’ lifestyle and thinking patterns. Neimeyer and Feixas [11] found that homework assignments that included records of thoughts, activity, and emotional responses alleviated depression in the short term for participants diagnosed with unipolar depression. Hundt et al [12] found that cognitive awareness of dysfunctional thoughts meditated the effectiveness of CBT treatments for depression. The literature demonstrates that mobile-based CBT is effective for reducing various psychological symptoms [13-15]; however, studies that examined standalone effects without professional contacts seemed to be limited [16]. To our knowledge, no other studies have assessed standalone effects of mobile CBT or any theory-based intervention.

### Mental Health Treatment in Japan

There are various reasons that people in Japan could benefit from CDIs. Many Japanese are unwilling to receive professional mental health services. Studies have shown that many Japanese view mental health problems as not a curable condition [17] and thus have little confidence in the effectiveness of treatment by mental health professionals. People may also avoid face-to-face mental health care in Japan because of societal stigmas [18]. Although advocating for receiving professional services for mental health problems is necessary for providing appropriate care, alternative forms of interventions such as CDIs may increase the accessibility of mental health services in Japan.

Recent research on Internet-based CBT has demonstrated reductions in depression among the Japanese [19,20]. People in the workforce are particularly vulnerable to psychological distress in Japan because overwork is common and predicts mental health problems [21]. To our knowledge, no studies have yet been conducted with CBT-based mobile interventions in Japan.

### Negative Mood Regulation Expectancies

One possible explanation of the effect of CBT-based interventions on psychological distress and substance use may be that CBT-based interventions increase individuals’ negative mood regulation expectancies. Negative mood regulation expectancies are defined as one’s belief that, when feeling upset, one can use thought or action to improve one’s mood [22]. Much research demonstrates that people with strong negative mood regulation expectancies cope more adaptively with stress and report fewer symptoms of anxiety and depression [23]. Negative mood regulation expectancies are also associated negatively with problematic drinking behavior, even after controlling for alcohol consumption, motivation, and coping styles [24]. Improvement in negative mood regulation expectancies during the early stages of CBT is associated with greater symptom reduction at the end of treatment and at follow-up [25,26]. Recent studies demonstrated that negative mood regulation expectancies operate in Japan in a similar way to how they do in the West [27,28].

### Development of a Mobile Phone-Delivered Cognitive Behavioral Therapy App

We developed an app called “ *jibun kiroku* ” [Self Record], which allows users to receive a CBT-based intervention on their mobile phone (see Multimedia Appendix 1). This app focuses particularly on self-monitoring and awareness of negative thoughts, daily activities, and daily mood. It first provides psychoeducation about the relationships among negative thoughts, feelings, behavior, and physical responses (see Multimedia Appendix 2). Users can record their daily activities on an hourly basis, which promotes cognitive restructuring by helping users to identify automatic thoughts, emotions, and behavior from their daily activities (see Multimedia Appendix 3). Users can also evaluate the quality of their sleep, mood, and energy level, and they can track changes over the week (see Multimedia Appendix 4).

### Study Purpose

The purpose of this study was to conduct a pilot trial to evaluate feasibility of the Self Record mobile phone app. We were particularly interested in examining standalone effects of the Self Record app on psychological distress and alcohol consumption. Although the intervention in this study was not developed to treat alcohol-related problems directly, we expected that reduction in psychological distress would lead to reduction in alcohol consumption, because alcohol consumption is positively associated with stress due to the tension-reducing properties of alcohol [29] and negatively with negative mood regulation expectancies [24]. We hypothesized that participants who received the intervention would show decreased psychological distress and alcohol consumption and increased psychological well-being and negative mood regulation expectancies (hypothesis 1). We also hypothesized that negative mood regulation expectancies would moderate the effect of the intervention on distress and alcohol consumption (hypothesis 2). Self-awareness, by itself, may not necessarily ameliorate symptoms; rather, people must have confidence that they can successfully regulate the processes they become aware of (ie, have strong negative mood regulation expectancies) [23].

## Methods

### Participation Selection

A research marketing company recruited individuals aged between 20 and 60 years, who indicated working full time and being “interested” or “somewhat interested” in using a self-monitoring app on their mobile phone. We also used a Japanese version of the Kessler Psychological Distress Scale (K6) to select participants experiencing psychological distress (see subsequent section for a full description of the K6). Participants whose K6 score fell between 5 and 12, indicating mild to moderate psychological distress, were recruited for this study. A total of 6921 participants were screened for eligibility, and 1396 met criteria for study participation.

### Outcome Measures

The Japanese Negative Mood Regulation Scale assessed negative mood regulation expectancies [27]. Forty items complete the stem: “When I’m mildly depressed or irritated...” Responses use a 5-point scale (1=strongly disagree to 5=strongly agree). An example item is “Telling myself it will pass will help me calm down.” Alphas in this study ranged from .91 to .93.

A Japanese version of the WHO (Five) Well-Being Index (WHO-5) assessed positive well-being [30]. The scale consists of five items that use a 6-point scale (0=at no time to 5=all of the time). An example item is “I felt cheerful and in good spirits.” The alphas in this study ranged from .89 to .92.

A Japanese version of the K6 was used to measure general distress [31]. The scale comprises six items that answer this question: “During the last 30 days, how did you feel about the following?” Examples items are “nervous” and “everything was an effort.” Participants use a 5-point scale (0=none of the time to 4=all of the time). Alphas in this study ranged from .84 to .85.

A Japanese version of the Center for Epidemiological Studies Depression Scale (CES-D) [32] measured depressive symptoms. The scale consists of 20 items responded to with a 4-point scale (0=rarely or none of the time [less than 1 day] to 3=most or all of the time [5-7 days]). The items assess various depressive symptoms (eg, “I feel sad,” “My sleep was restless,” “I felt that people dislike me”). Alphas for this study ranged from .84 to .89.

Trait anxiety was assessed using the State-Trait Anxiety Inventory (STAI-Trait). A Japanese version was created by Shimizu and Imae [33]. The scale consists of 20 items responded to with a 4-point scale (1=almost never to 4=almost always). Items include “I lack self-confidence,” “I worry over something that doesn’t matter,” and “I feel secure.” Alphas in this study ranged from .88 to .89.

To assess typical drinking and heavy drinking in the last 30 days, the Daily Drinking Questionnaire was used [34]. For typical drinking, participants recall a typical week and indicate how many drinks they consumed and the time they spent drinking each day from Monday to Sunday. For heavy drinking, they recalled the week they drank the most alcohol. A sum score of typical drinking and heavy drinking was calculated by summing the drinking quantity of all seven days. Participants reported their drinking quantity by converting all their drinks to 500 mL of beer, which is equivalent to 1 unit of alcohol in Japan.

### Procedure

The Life Science Research Ethics and Safety committee at the University of Tokyo in Tokyo, Japan, reviewed and approved this study (16-88). Participants, who were registered in the research marketing company’s pool, received a notification about the study over the Internet. The screening for eligibility asked potential participants whether they were willing to use a self-monitoring app on their mobile phone. Specifics about the app were not revealed until after participants consented to be in the study. Because this study had to provide the intervention to those who were interested in using the app, this study implemented a quasi-experimental design. Participants who were willing to use the Self Record app represented the intervention condition, and those who were not represented the control condition.

After participants were screened for eligibility, they read the informed consent and then completed pretest measures in this order: general distress, negative mood regulation expectancies, depression, anxiety, alcohol consumption, and positive well-being. After the 4-week intervention period, participants in both conditions retook the identical battery of questionnaires.

### Intervention

Participants in the intervention condition first read a psychoeducation section presenting the basic concepts of CBT, which included an explanation about how thoughts affect feelings, behavior, and physical reactions (see Multimedia Appendix 2). Then, they were instructed to record their daily activities, thoughts, mood, and sleep quality, which would help them discover and modify dysfunctional thoughts as part of cognitive restructuring (see Multimedia Appendixes 3 and 4). To identify the frequency of their app use, they were instructed to send a screenshot of their weekly daily activity and thought records at the beginning of the trial period. All participation was conducted online.

### Experimental Design and Statistical Analyses

All participants in the intervention and control groups were included for intention-to-treat analyses. Multiple imputations were performed to replace missing values that were lost at follow-up. Additionally, we performed per-protocol analyses because some variation in app use was expected and that inaccurate responses in outcome measures indicate lack of diligence.

For examining the effectiveness of the intervention (hypothesis 1), we treated the condition (intervention vs control), the time (pretest and posttest), and the condition × time interaction as independent variables. Dependent variables were general distress (K6), depression (CES-D), anxiety (STAI-Trait), positive well-being (WHO-5), negative mood regulation expectancies, typical drinking, and heavy drinking. Mixed-effect analyses of variance (ANOVA) were performed. The condition × time interaction examined the effect of using the intervention for 4 weeks compared to the assessment-only effect in the control condition. An R package called “nlme” was used to perform this analysis [35].

To examine the moderating effect of negative mood regulation expectancies (hypothesis 2), we implemented multiple regression analyses. Independent variables were condition (intervention vs control), negative mood regulation expectancies, and the condition × negative mood regulation expectancies interaction. For dependent variables, we used difference in the outcome variables between posttest and the pretest to simplify the statistical analyses. An R package called “car” was used to perform the multiple regression analyses [36]. For effect sizes, we calculated beta. All analyses used the .05 level of significance.

### Power Analysis

Based on prior studies’ weak effect sizes for CDIs on alcohol consumption, we estimated at least a sample of 393 was needed for 80% statistical power at the *P*<.05 significance level. Because high attrition and poor quality of responses were expected, we collected beyond the number estimated by the power analysis.

## Results

### Attrition

**Figure 1:**
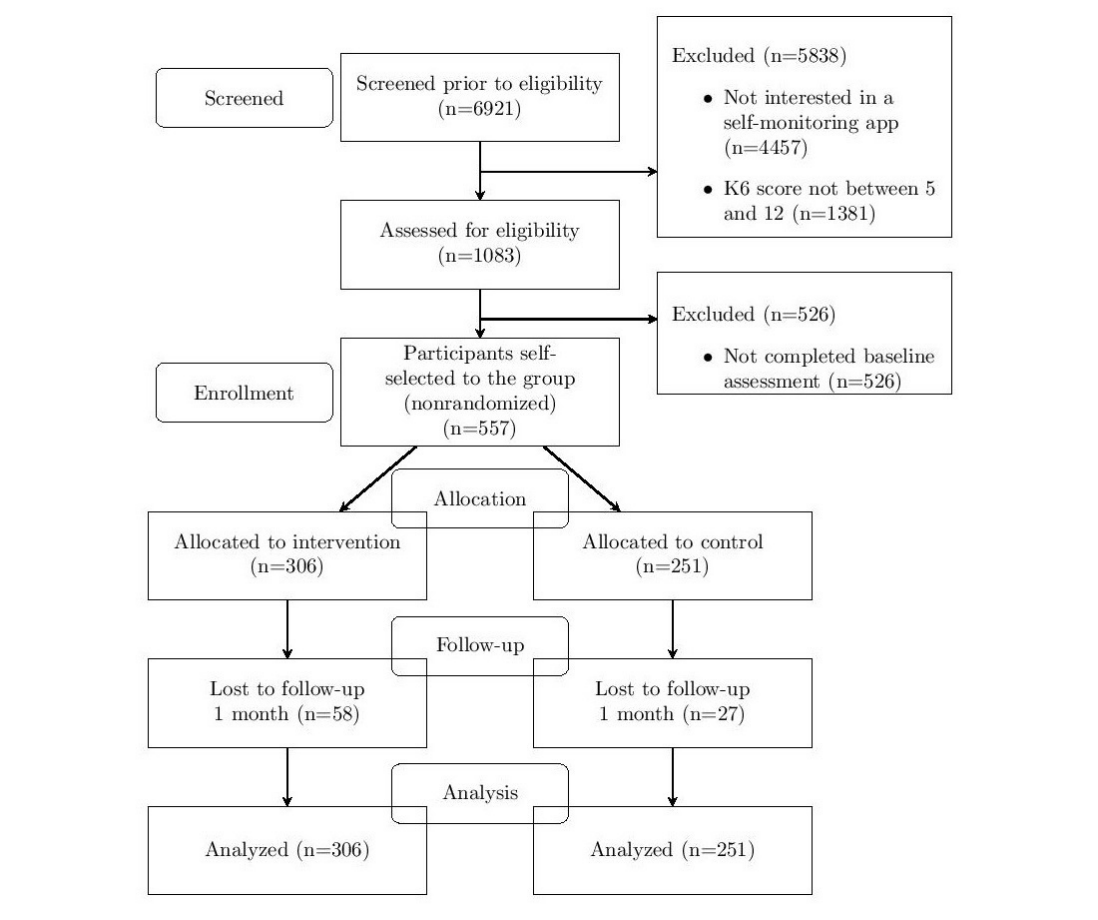
The Consolidated Standards of Reporting Trials (CONSORT) extension flow diagram.

Figure 1 shows the flowchart of this study. During recruitment beginning in February 2017, 6921 participants were approached for screening, 1083 of 6921 (15.65%) met eligibility criteria, and 557 of 6921 (8.05%) completed the baseline measure. Then, 306 of 557 participants (54.9%) were allocated to the intervention group and 251 of 557 (45.1%) to the control group based on their self-selection regarding willingness to use the mobile phone app. The 1-month follow-up occurred in March 2017; 58 of 306 participants (19.0%) in the intervention group and 27 of 251 (10.8%) participants in the control group were lost because they did not complete outcome measures.

For the frequency of the Self Record app use, 107 of 306 participants (35.0%) reported they continued using the app after the first day, 63 participants (20.6%) used it for more than a week, 37 participants (12.1%) used it for more than 2 weeks, 14 participants (4.6%) used it for more than 3 weeks, and 8 participants (2.6%) used it for more than 4 weeks.

### Participant Characteristics

Of the 557 participants enrolled in the trial, 230 (41.2%) were women. The mean age was 38.82 (SD 9.58) years. Regarding work status, 399 of 557 (71.6%) reported employment by a company, 42 of 557 (7.5%) reported employment by the government or a nonprofit organization, 35 of 557 (6.3%) reported self-employment, and 17 of 557 (3.1%) reported working as a professional (eg, lawyer, accountant, and medical staff).

### Data Screening

Data were screened for missingness, outliers, normality, and heteroscedasticity. All measures were winsorized to adjust outliers. For the K6, CES-D, and WHO-5, square-root transformation was used, and for typical drinking and heavy drinking, logarithmic transformation was used to adjust for positive skewness and kurtosis.

### Descriptive Statistics

Descriptive statistics are shown in Table 1. Multivariate analysis of variance indicated no significant group differences in the pretest variables (*F*_7,549_=0.95, *P=*.47). The mean scores on the K6 and CES-D were higher than in other studies that examined Japanese workers [19,20], indicating higher levels of psychological distress among participants in this study.

### Intention-to-Treat Primary Analyses

**Figure 2:**
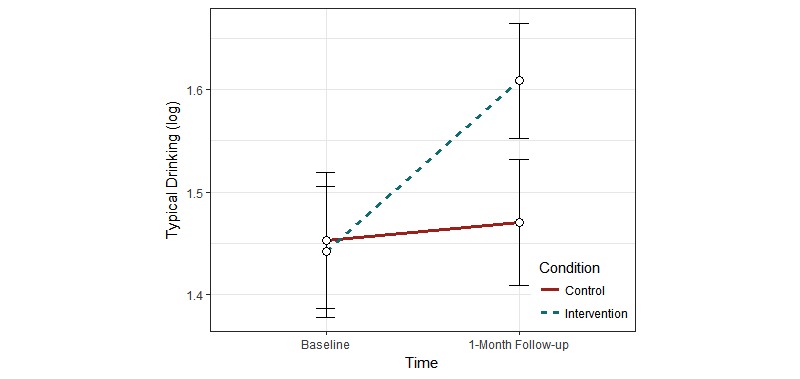
Change in typical drinking as a function of condition and time in the intention-to-treat analysis.

**Figure 3:**
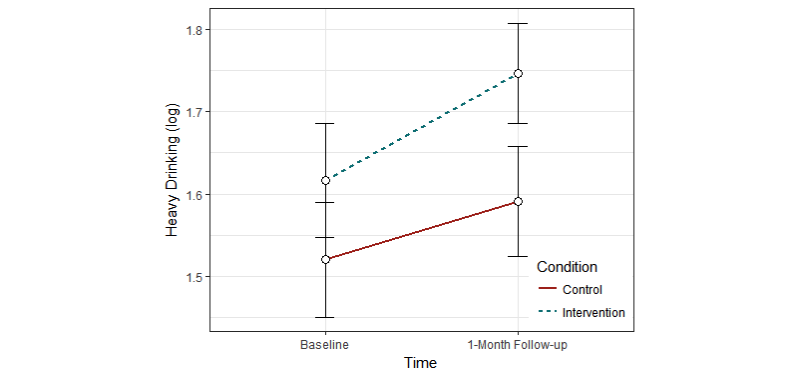
Change in heavy drinking as a function of condition and time in the intention-to-treat analysis.

For intention-to-treat analyses, mixed-effect ANOVA was used to assess the main effects of condition and time, and the condition × time interaction (hypothesis 1). Table 2 shows results of the analyses. The main effect of condition was significant for typical drinking (η^2^<.001) and heavy drinking (η^2^=.002). Participants in the intervention group reported higher typical and heavy drinking than did those in the control group. The main effect of time was significant for WHO-5 (η^2^=.003), negative mood regulation expectancies (η^2^=.004), K6 (η^2^=.002), CES-D (η^2^=.003), typical drinking (η^2^<.001), and heavy drinking (η^2^=.002). At the 1-month follow-up, participants reported higher scores on the WHO-5, K6, CES-D, typical drinking, and heavy drinking, and lower negative mood regulation expectancies compared to baseline. The condition × time interaction was significant for typical drinking (η^2^=.009; see Figure 2) and heavy drinking (η^2^=.001; see Figure 3). For both typical and heavy drinking, participants in the intervention group reported higher scores at the 1-month follow-up compared to the participants in the control group.

We also examined the moderation by negative mood regulation expectancies of the effect of the intervention (hypothesis 2). Because three-way interactions are often difficult to interpret, we implemented multiple regression analyses. Independent variables were group (intervention and control), negative mood regulation expectancies, and the group × negative mood regulation expectancies interaction. Dependent variables were changes from baseline to 1-month follow-up of outcome variables. None of the results were statistically significant.

**Table 1:** Mean and standard deviation of outcome measures (N=557).

Test^a^		Control, mean (SD)	Intervention, mean (SD)
**NMRE**			
	Pretest	120.86 (12.59)	120.84 (14.70)
	Posttest	119.66 (12.60)	118.58 (15.73)
**WHO-5**			
	Pretest	14.25 (4.91)	14.65 (5.23)
	Posttest	14.59 (4.73)	15.18 (5.25)
**K6**			
	Pretest	7.40 (4.27)	7.43 (4.80)
	Posttest	7.98 (4.76)	8.08 (4.75)
**CES-D**			
	Pretest	19.44 (7.75)	19.62 (8.81)
	Posttest	20.21 (8.24)	21.59 (9.32)
**STAI-Trait**			
	Pretest	49.83 (7.30)	49.91 (8.29)
	Posttest	49.57 (6.80)	50.53 (8.64)
**Typical drinking**			
	Pretest	6.15 (7.63)	6.70 (8.93)
	Posttest	5.67 (6.16)	6.60 (6.77)
**Heavy drinking**			
	Pretest	6.96 (8.63)	8.89 (12.03)
	Posttest	6.94 (7.51)	8.12 (8.21)

^a^CES-D: Centers for Epidemiological Studies Depression; K6: Kessler Psychological Distress Scale; NMRE: Negative mood regulation expectancies; STAI: State-Trait Anxiety Inventory; WHO-5: WHO (Five) Well-Being Index.

**Table 2:** Intention-to-treat primary analyses: effects of condition, time, and condition × time interaction on outcome variables.

Test^a^	Condition			Time			Condition × time		
	χ^2^_5_	*P*	η^2^	χ^2^_6_	*P*	η^2^	χ^2^_7_	*P*	η^2^
NMRE	0.3	.61	<.001	10.7	.001	.004	1.0	.33	.002
WHO-5	1.4	.23	.001	5.0	.03	.003	0.1	.71	.006
K6	0.02	.89	<.001	5.9	.02	.002	0.6	.45	.01
CES-D	0.7	.40	<.001	14.2	<.001	.003	2.8	.10	.03
STAI-Trait	1.3	.52	<.001	0.5	.47	<.001	2.5	.29	.004	
Typical drinking	9.5	.009	<.001	8.9	.003	<.001	13.9	.001	.009
Heavy drinking	10.1	.006	.002	8.0	.003	.002	8.7	.01	.001

^a^CES-D: Centers for Epidemiological Studies Depression; K6: Kessler Psychological Distress Scale; NMRE: Negative mood regulation expectancies; STAI: State-Trait Anxiety Inventory; WHO-5: WHO (Five) Well-Being Index.

### Per-Protocol Secondary Analyses

The intention-to-treat analyses were problematic due to attrition; approximately two-thirds of the participants (65.0%, 199/306) in the intervention group discontinued using the app on the first day. In addition, two-thirds of the participants in both groups (65.7%, 366/557) incorrectly answered validity items. As a result, substantially lower alphas for outcome measures were observed when these participants were included in reliability analyses, suggesting random or inattentive responding. For these reasons, we conducted per-protocol analyses by excluding participants who discontinued using the app after the first day and answered the validity items incorrectly. Forty-two participants in the intervention and 85 participants in the control remained in the analyses.

Table 3 shows results of the per-protocol secondary analyses. A mixed-effect ANOVA revealed that the main effect of condition was significant for K6 (η^2^=.09). Participants in the intervention group reported significantly lower K6 scores than did those in the control group. The main effect of time was significant for negative mood regulation expectancies (η^2^=.009). Participants reported significantly lower negative mood regulation expectancies at the 1-month follow-up. The condition × time interaction was significant for anxiety (η^2^=.04), typical drinking (η^2^=.06), and heavy drinking (η^2^=.09). Participants in the intervention group reported higher scores for all three outcome measures at the 1-month follow-up compared to the control group.

**Table 3:** Per-protocol secondary analyses: effects of condition, time, and condition × time interaction on outcome variables.

Test^a^	Condition			Time			Condition × time		
	χ^2^_5_	*P*	η^2^	χ^2^_6_	*P*	η^2^	χ^2^_7_	*P*	η^2^
NMRE	1.4	.23	.02	4.6	.03	.009	1.5	.22	.01
WHO-5	1.4	.24	.01	0.2	.65	<.001	0.01	.92	<.001
K6	12.2	<.001	.09	0.7	.41	.009	0.5	.47	.04
CES-D	2.0	.16	.03	0.006	.94	.003	1.4	.23	.008
STAI-Trait	3.9	.14	.05	1.1	.29	.002	6.7	.04	.04	
Typical drinking	0.4	.81	<.001	0.1	.82	.02	7.4	.03	.06
Heavy drinking	1.0	.60	<.001	0.2	.69	.04	12.2	.002	.09

^a^CES-D: Centers for Epidemiological Studies Depression; K6: Kessler Psychological Distress Scale; NMRE: Negative mood regulation expectancies; STAI: State-Trait Anxiety Inventory; WHO-5: WHO (Five) Well-Being Index.

For moderation by negative mood regulation expectancies (hypothesis 2), the condition × negative mood regulation expectancies interaction predicted general distress (beta=.39, *P*=.03). Among participants with high negative mood regulation expectancies, those in the intervention group reported higher general distress compared to those in the control group. However, there was no difference among those with low negative mood regulation expectancies.

## Discussion

This study examined standalone effects of a CBT-based mobile intervention called Self Record on psychological distress and drinking consumption. The intention-to-treat analyses revealed that participants who used the Self Record app reported increased typical drinking and heavy drinking, which was contrary to our hypothesis. In the per-protocol secondary analyses, we removed participants who discontinued using the app on the first day and participants who showed inattentiveness in answering outcome measures. The per-protocol analysis revealed that participants who used the intervention more often over the 4-week trial period reported increases in anxiety, typical drinking, and heavy drinking compared to those who rarely used the intervention or members of the control group. The effect sizes were small to medium.

The goal of the intervention in this study was to raise participants’ awareness of potentially pathological thinking and to discover solutions to it. The first objective may have succeeded, raising participants’ awareness of their maladaptive patterns; however, the intervention apparently did not help participants self-treat their problematic thinking. Consequently, this heightened awareness may have affected results in two ways: (1) people may have reported increases in distress because they became more aware of their distress, and (2) such awareness may have started a vicious cycle in which heightened awareness led to greater distress, which the interventions focused their awareness on.

Our result was contrary to previous studies that demonstrated the positive effects of mobile phone-based interventions on psychological distress [14,15]. One difference of these studies’ designs is that their participants had personal contacts with a mental health professional or researcher to discuss their experience with the interventions, whereas participants in this study focused on self-monitoring and had no personal contacts with any professional. It is possible that contact with the professional was more responsible for symptom improvement than was the CDI in prior studies. Gajecki et al [37] investigated a standalone mobile-based intervention for reducing risky drinking. They also found that using the mobile app led to more frequent alcohol use among men. Standalone use of CDIs was completely reliant on the participants for its success. Our findings and those of Gajecki et al highlight that, if participants are not capable of successfully using the information the intervention provides, the intervention may have unintended negative consequences.

We also investigated the moderating role of negative mood regulation expectancies on the effect of the intervention. The level of negative mood regulation expectancies affected the impact of the intervention on general distress. Among participants with high negative mood regulation expectancies, those in the control condition showed decreases, whereas those in the intervention condition showed increases in general distress. The unexpected effects of the Self Record app on general distress were more evident among those with high negative mood regulation expectancies than those with low negative mood regulation expectancies. More studies are necessary to investigate the moderating role of negative mood regulation expectancies on CDI psychological interventions.

There are several limitations of this study. First, this study used a quasi-experimental design, in which only those who were interested in using the mobile phone-based intervention were placed in the intervention group. Although there were no group differences in measures at pretest, it is still possible that some unmeasured factor was responsible for changes from pretest to posttest. More research needs to be conducted to confirm the findings of this study. Confounding variables, such as participants’ willingness to use the intervention or other characteristics, might have contributed to the findings in this study. Also, the study was conducted during the last month of the fiscal year in Japan and the participants, who were all full-time workers, may have been experiencing workplace stress. This environmental factor may have contributed in the increase in their stress in general and may have overshadowed the influence of personality variables.

Second, there was a high attrition rate in this study. Although attrition at 1-month follow-up was not relatively high (15.3%, 85/557), 65.0% (199/306) of the participants in the intervention group discontinued using the app on the first day. Furthermore, 65.7% (366/557) of the total sample incorrectly answered all the accuracy-check items. Some literature points to high attrition as a characteristic of many Internet-based studies [38], but this attrition may limit the generalizability of our results.

Nevertheless, this study may suggest unintended effects of standalone use of mobile-based CDIs for Japanese experiencing moderate stress. The mobile intervention may be beneficial when it is combined with other interventions that include contact with professionals to discuss thoughts and behaviors. This contact would allow clinicians to adjust interventions that lead countertherapeutically to higher distress. More intense computerized interventions or face-to-face psychotherapy may be necessary to alleviate psychological stress.

Future studies may use more rigorous research designs, such as a randomized controlled trial. If standalone computerized self-monitoring increases people’s anxiety and drinking behavior, future studies may clarify mechanisms for such increases. There are several ways to improve low response rates. First, reminders can be sent to participants about using the app because this study did not provide any reminders. Second, participants could receive more specific instructions for using the app, so that they could focus on the daily activities and negative thoughts they prefer to work on with the app. This will focus their thinking on the most important thoughts for intervention. Third, future studies should design apps with more automatic functions, such as syncing with other apps to record sleep time, to reduce the manual entries needed to be done by users.

## Appendix

Multimedia Appendix 1 A screenshot of the download and the home screen.

Multimedia Appendix 2 A screenshot of the psychoeducation component.

Multimedia Appendix 3 A screenshot of the thought and behavior records.

Multimedia Appendix 4 A screenshot of the mood records.

## Abbreviations

CBT: cognitive behavioral therapy
CDI: computer-delivered intervention
CES-D: Centers for Epidemiological Studies Depression
K6: Kessler Psychological Distress Scale
STAI: State-Trait Anxiety Inventory
WHO-5: WHO (Five) Well-Being Index

## References

[1] Andersson G (2016). Internet-delivered psychological treatments. Annu Rev Clin Psychol.

[2] Richards D, Richardson T (2012). Computer-based psychological treatments for depression: a systematic review and meta-analysis. Clin Psychol Rev.

[3] Spek V, Cuijpers P, Nyklícek I, Riper H, Keyzer J, Pop V (2007). Internet-based cognitive behaviour therapy for symptoms of depression and anxiety: a meta-analysis. Psychol Med.

[4] Dedert EA, McDuffie JR, Stein R, McNiel JM, Kosinski AS, Freiermuth CE, Hemminger A, Williams JW (2015). Electronic interventions for alcohol misuse and alcohol use disorders: a systematic review. Ann Intern Med.

[5] Donker T, Petrie K, Proudfoot J, Clarke J, Birch M, Christensen H (2013). Smartphones for smarter delivery of mental health programs: a systematic review. J Med Internet Res.

[6] Lindhiem O, Bennett CB, Rosen D, Silk J (2015). Mobile technology boosts the effectiveness of psychotherapy and behavioral interventions: a meta-analysis. Behav Modif.

[7] Watts S, Mackenzie A, Thomas C, Griskaitis A, Mewton L, Williams A, Andrews G (2013). CBT for depression: a pilot RCT comparing mobile phone vs computer. BMC Psychiatry.

[8] Gustafson DH, McTavish FM, Chih M, Atwood AK, Johnson RA, Boyle MG, Levy MS, Driscoll H, Chisholm SM, Dillenburg L, Isham A, Shah D (2014). A smartphone application to support recovery from alcoholism: a randomized clinical trial. JAMA Psychiatry.

[9] Butler AC, Chapman JE, Forman EM, Beck AT (2006). The empirical status of cognitive-behavioral therapy: a review of meta-analyses. Clin Psychol Rev.

[10] Windsor LC, Jemal A, Alessi EJ (2015). Cognitive behavioral therapy: a meta-analysis of race and substance use outcomes. Cultur Divers Ethnic Minor Psychol.

[11] Neimeyer RA, Feixas G (1990). The role of homework and skill acquisition in the outcome of group cognitive therapy for depression. Behav Ther.

[12] Hundt NE, Mignogna J, Underhill C, Cully JA (2013). The relationship between use of CBT skills and depression treatment outcome: a theoretical and methodological review of the literature. Behav Ther.

[13] Ly KH, Trüschel A, Jarl L, Magnusson S, Windahl T, Johansson R, Carlbring P, Andersson G (2014). Behavioural activation versus mindfulness-based guided self-help treatment administered through a smartphone application: a randomised controlled trial. BMJ Open.

[14] Kauer SD, Reid SC, Crooke AHD, Khor A, Hearps SJC, Jorm AF, Sanci L, Patton G (2012). Self-monitoring using mobile phones in the early stages of adolescent depression: randomized controlled trial. J Med Internet Res.

[15] Morris ME, Kathawala Q, Leen TK, Gorenstein EE, Guilak F, Labhard M, Deleeuw W (2010). Mobile therapy: case study evaluations of a cell phone application for emotional self-awareness. J Med Internet Res.

[16] Fitzpatrick KK, Darcy A, Vierhile M (2017). Delivering cognitive behavior therapy to young adults with symptoms of depression and anxiety using a fully automated conversational agent (Woebot): a randomized controlled trial. JMIR Ment Health.

[17] Ando S, Yamaguchi S, Aoki Y, Thornicroft G (2013). Review of mental-health-related stigma in Japan. Psychiatry Clin Neurosci.

[18] Desapriya EB, Nobutada I (2002). Stigma of mental illness in Japan. Lancet.

[19] Imamura K, Kawakami N, Furukawa TA, Matsuyama Y, Shimazu A, Umanodan R, Kawakami S, Kasai K (2014). Effects of an Internet-based cognitive behavioral therapy (iCBT) program in Manga format on improving subthreshold depressive symptoms among healthy workers: a randomized controlled trial. PLoS One.

[20] Kojima R, Fujisawa D, Tajima M, Shibaoka M, Kakinuma M, Shima S, Tanaka K, Ono Y (2010). Efficacy of cognitive behavioral therapy training using brief e-mail sessions in the workplace: a controlled clinical trial. Ind Health.

[21] Yamauchi T, Yoshikawa T, Takamoto M, Sasaki T, Matsumoto S, Kayashima K, Takeshima T, Takahashi M (2017). Overwork-related disorders in Japan: recent trends and development of a national policy to promote preventive measures. Ind Health.

[22] Catanzaro SJ, Mearns J (1990). Measuring generalized expectancies for negative mood regulation: initial scale development and implications. J Pers Assess.

[23] Catanzaro S, Mearns J, Trusz S, Babel P (2016). Generalized expectancies for negative mood regulation: development, assessment, and implications of a construct. Intrapersonal and Interpersonal Expectancies: Research, Applications and Future Directions.

[24] Kassel JD, Jackson SI, Unrod M (2000). Generalized expectancies for negative mood regulation and problem drinking among college students. J Stud Alcohol.

[25] Backenstrass M, Schwarz T, Fiedler P, Joest K, Reck C, Mundt C, Kronmueller K (2006). Negative mood regulation expectancies, self-efficacy beliefs, and locus of control orientation: moderators or mediators of change in the treatment of depression?. Psychother Res.

[26] Cloitre M, Stovall-McClough KC, Nooner K, Zorbas P, Cherry S, Jackson CL, Gan W, Petkova E (2010). Treatment for PTSD related to childhood abuse: a randomized controlled trial. Am J Psychiatry.

[27] Mearns J, Self E, Kono K (2016). Measuring generalized expectancies for negative mood regulation in Japan: the Japanese language NMR Scale. Int J Quant Res Educ.

[28] Hamamura T, Mearns J (2017). Depression and somatic symptoms in Japanese and American college students: negative mood regulation expectancies as a personality correlate. Int J Psychol.

[29] Greeley J, Oei T, Leonard K, Blane H (1999). Alcohol and tension reduction. Psychological Theories of Drinking and Alcoholism.

[30] Awata S, Bech P, Koizumi Y, Seki T, Kuriyama S, Hozawa A, Ohmori K, Nakaya N, Matsuoka H, Tsuji I (2007). Validity and utility of the Japanese version of the WHO-Five Well-Being Index in the context of detecting suicidal ideation in elderly community residents. Int Psychogeriatr.

[31] Furukawa TA, Kawakami N, Saitoh M, Ono Y, Nakane Y, Nakamura Y, Tachimori H, Iwata N, Uda H, Nakane H, Watanabe M, Naganuma Y, Hata Y, Kobayashi M, Miyake Y, Takeshima T, Kikkawa T (2008). The performance of the Japanese version of the K6 and K10 in the World Mental Health Survey Japan. Int J Methods Psychiatr Res.

[32] Shima S, Shikano T, Kitamura T, Asai M (1985). A new self-rating scales for depression [in Japanese]. Seishin Igaku.

[33] Shimizu H, Imae K (1981). Development of a Japanese version of the State-Trait Anxiety inventory for college students [in Japanese]. Kyoiku shinrigaku kenkyu.

[34] Collins RL, Parks GA, Marlatt GA (1985). Social determinants of alcohol consumption: the effects of social interaction and model status on the self-administration of alcohol. J Consult Clin Psychol.

[35] Pinheiro J, Bates D, DebRoy S, Sarkar D, Heisterkamp S, Van Willigen B, EISPACK authors (2017). Linear and nonlinear mixed effects models.

[36] Fox J, Weisberg S, Adler D, Bates D, Baud-Bovy G, Ellison S, Firth D, Friendly M, Gorjanc G, Graves S, Heiberger R, Laboissiere R, Monette G, Murdoch D, Nilsson H, Ogle D, Ripley B, Venables W, Winsemius D, Zeileis A, R-Core (2017). Companion to applied regression.

[37] Gajecki M, Berman AH, Sinadinovic K, Rosendahl I, Andersson C (2014). Mobile phone brief intervention applications for risky alcohol use among university students: a randomized controlled study. Addict Sci Clin Pract.

[38] Eysenbach G (2005). The law of attrition. J Med Internet Res.

