# Supplementary figures and images for: Standalone Effects of a Cognitive Behavioral Intervention Using a Mobile Phone App on Psychological Distress and Alcohol Consumption Among Japanese Workers: Pilot Nonrandomized Controlled Trial
Source: JMIR Ment Health. 2018 Mar 22;5(1):e24. doi: 10.2196/mental.8984 (PMC5887038; doi:10.2196/mental.8984)

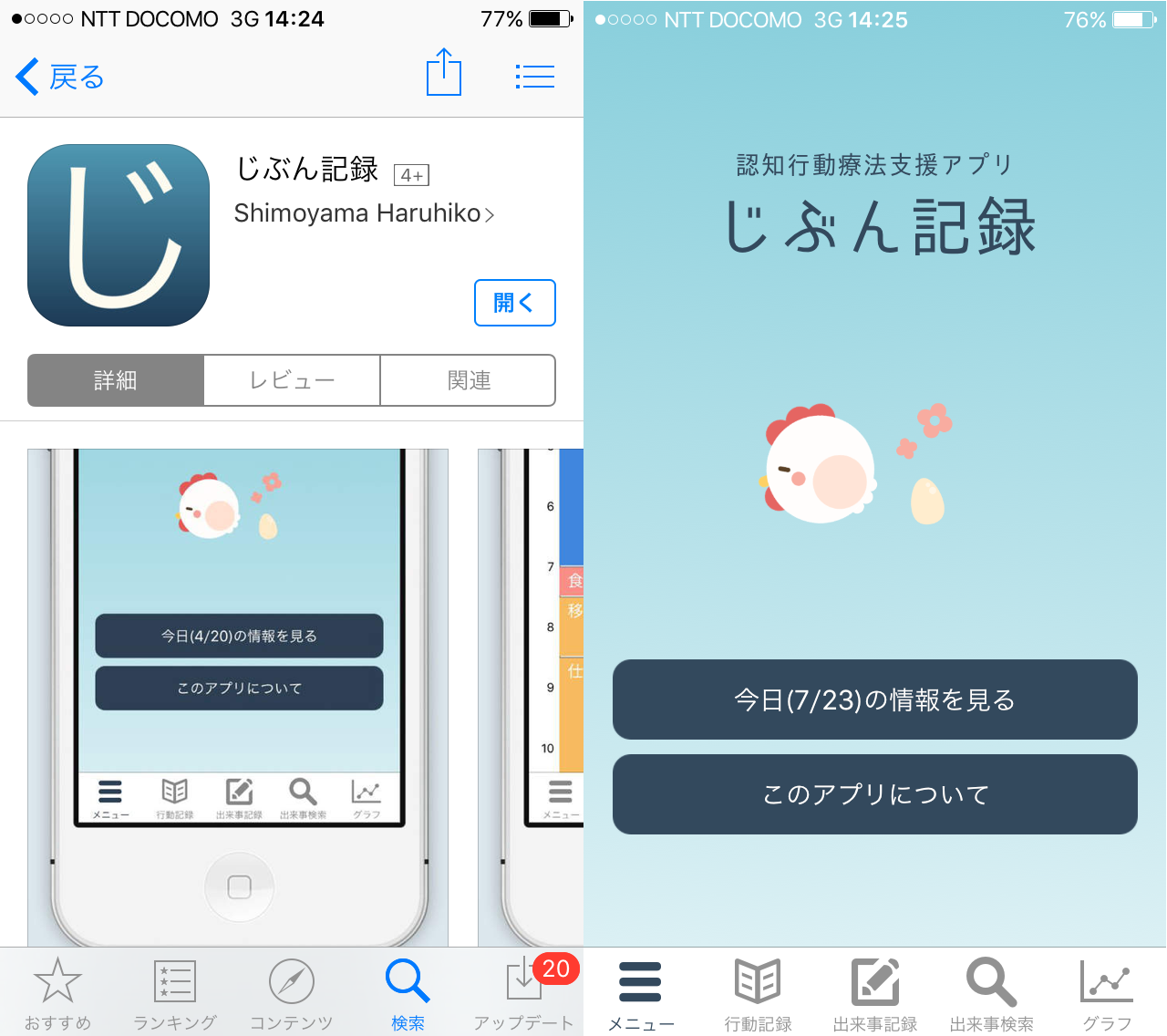

Supplement: Multimedia Appendix 1 [file mental_v5i1e24_app1.png]

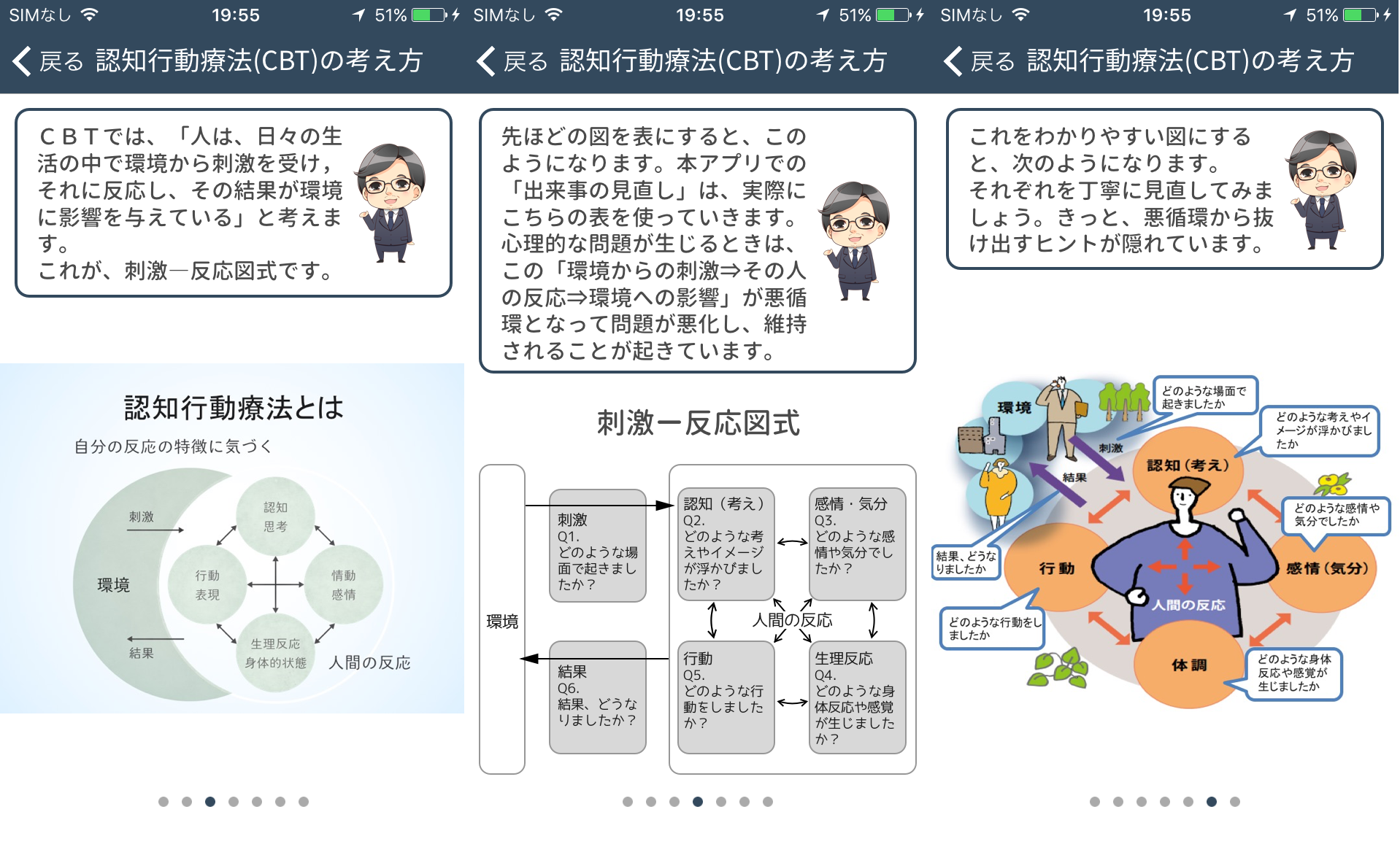

Supplement: Multimedia Appendix 2 [file mental_v5i1e24_app2.png]

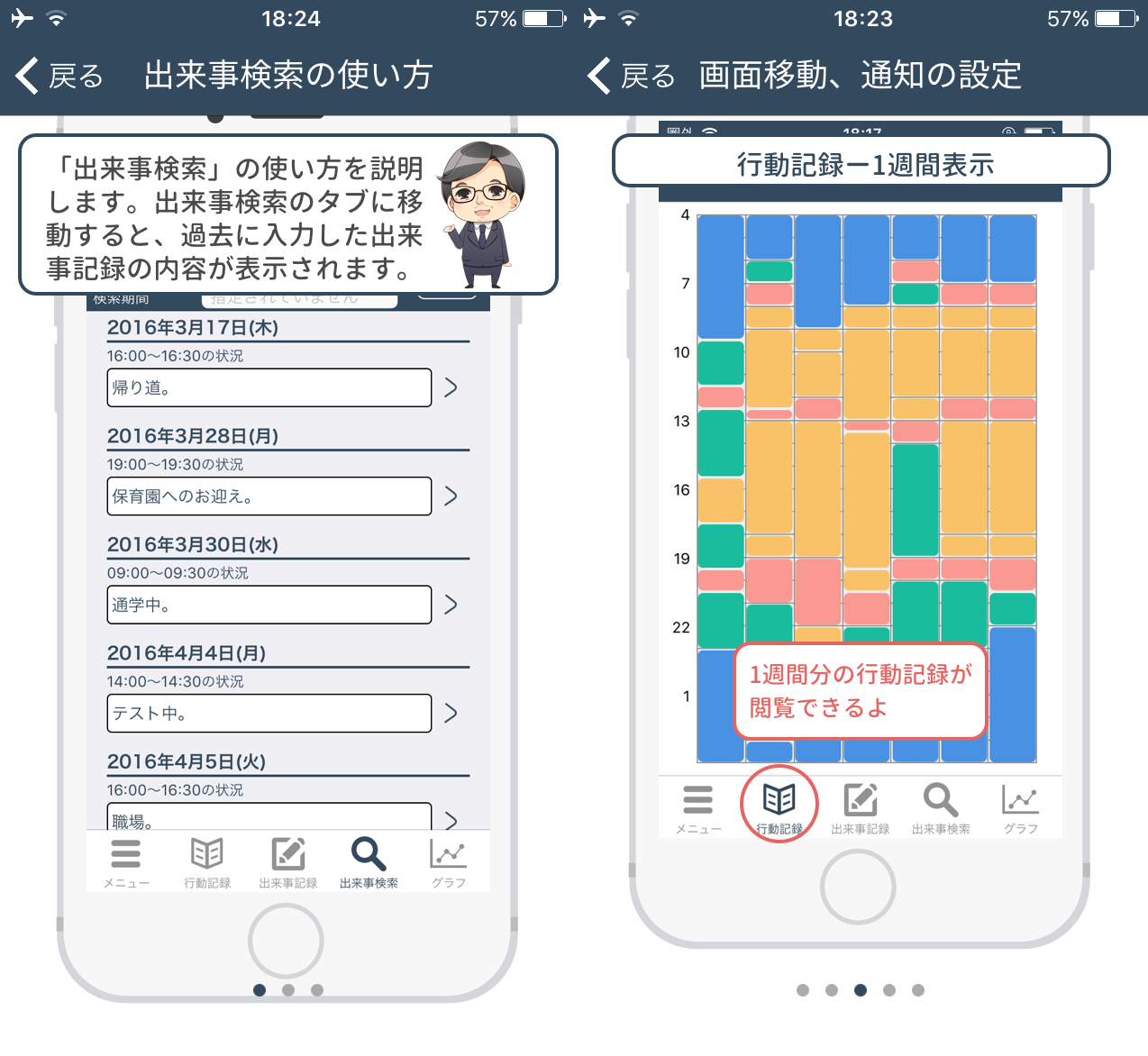

Supplement: Multimedia Appendix 3 [file mental_v5i1e24_app3.png]

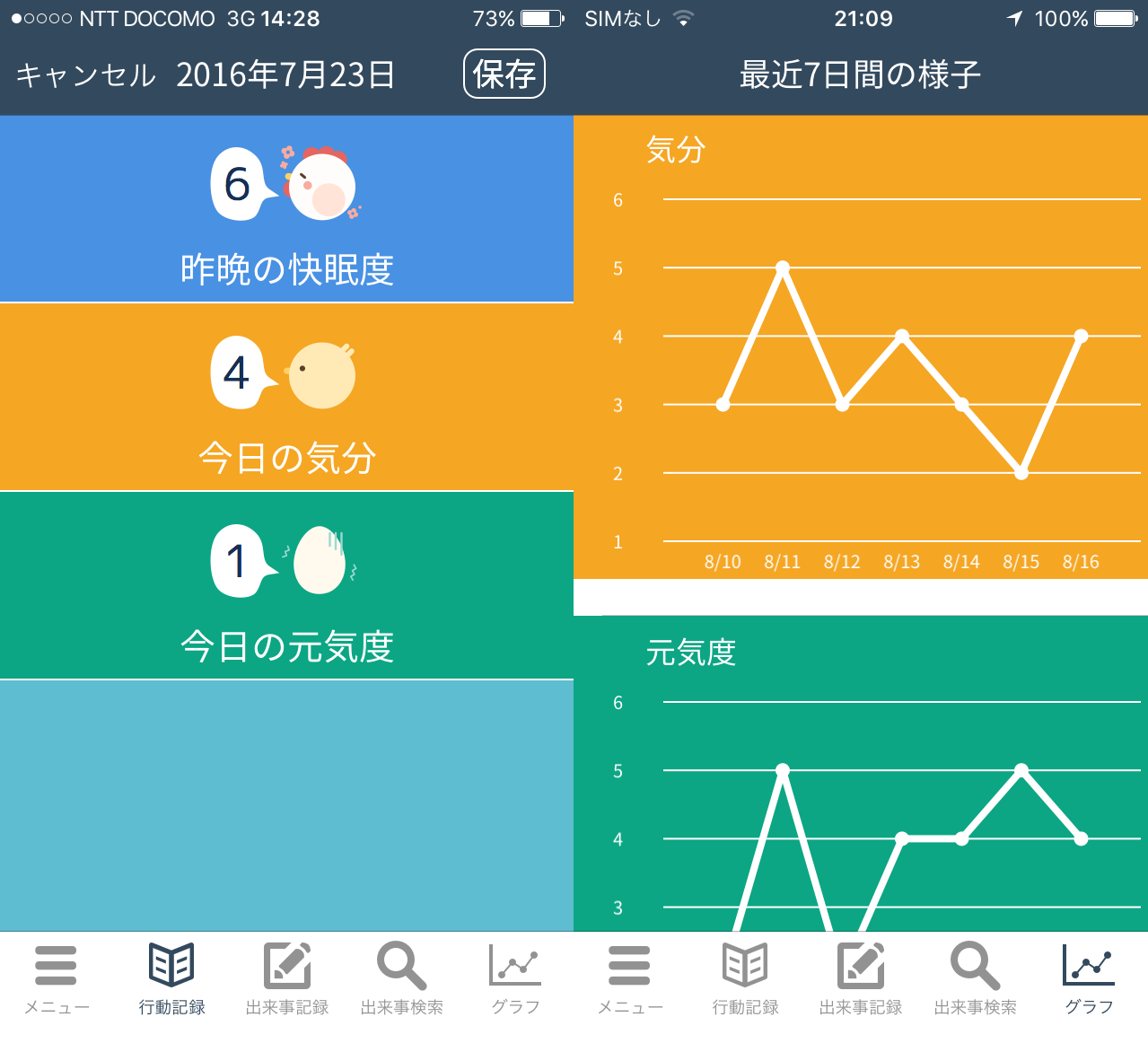

Supplement: Multimedia Appendix 4 [file mental_v5i1e24_app4.png]
